# Citizen Worry and Adherence in Response to Government Restrictions in Switzerland During the COVID-19 Pandemic: Repeated Cross-Sectional Online Surveys

**DOI:** 10.2196/55636

**Published:** 2025-01-07

**Authors:** Vanessa Kraege, Céline Dumans-Louis, Céline Maglieri, Séverine Bochatay, Marie-Anne Durand, Antoine Garnier, Kevin Selby, Christian von Plessen

**Affiliations:** 1 Innovation and Clinical Research Directorate University Hospital of Lausanne Lausanne Switzerland; 2 Faculty of Biology and Medicine University of Lausanne Lausanne Switzerland; 3 Department of Internal Medicine Le Groupement Hospitalier de l’Ouest Lémanique 1260 Nyon Switzerland; 4 Faculty of Arts University of Lausanne Lausanne Switzerland; 5 Department of Ambulatory Care Centre universitaire de médecine générale et santé publique, Lausanne Lausanne Switzerland; 6 The Dartmouth Institute for Health Policy and Clinical Practice Dartmouth College Hanover, NH United States; 7 Faculty of Science and Medicine University of Fribourg Fribourg Switzerland; 8 Department of Medicine and Specialities Fribourg Cantonal Hospital Fribourg Switzerland

**Keywords:** COVID-19 pandemic, citizens, worry, anxiety, communication, prevention, adherence, restrictions, Switzerland, cross sectional, online survey, survey, Swiss, adults, questionnaire, social media, linear regression, age, gender, health literacy, education, women, young people

## Abstract

**Background:**

Good communication between health authorities and citizens is crucial for adherence to preventive measures during a pandemic. Crisis communication often appeals to worries about negative consequences for oneself or others. While worry can motivate protective behavior, it can also be overwhelming and lead to irrational choices or become a mental health problem. Also, the levels and consequences of worry can differ between different groups of citizens. Little is known about the evolution of worries during the pandemic and adherence to measures in distinct groups.

**Objective:**

This study aimed to evaluate worries in the Swiss population as well as associations between worry levels and citizens’ adherence to government restrictions during different phases of the COVID-19 pandemic.

**Methods:**

We carried out an observational study with 4 cross-sectional online surveys of adults in the Canton of Vaud, Switzerland. Questionnaires were distributed through social media and websites during 4 periods: survey 1: April 17 to May 14, 2020; survey 2: May 15 to June 22, 2020; survey 3: October 30 to December 12, 2020; and survey 4: June 18 to December 30, 2021. On visual analog scales from 0 to 100, participants reported worry, self-adherence to pandemic restrictions, and their perceived adherence to others. We used multivariable linear regression, adjusting for age, gender, health literacy, and education to assess associations between self-reported worry, adherence, and study periods.

**Results:**

We collected 7106 responses. After excluding 2377 questionnaires (incomplete, age <18 years, residence outside Vaud), 4729 (66.55%) were analyzed (mean age 47, SD 15.6 years, 63.96% women). Mean worry across the 4 periods was 42/100, significantly higher in women (44.25/100, vs 37.98/100; *P*<.001) and young people (43.77/100 in those aged 18-39 years, vs 41.69/100; *P*=.005; in those aged 40-64 years and 39.16/100; *P*=.002; in those aged >64 years). Worries were higher during survey 1 and survey 3 (52.41/100 and 56.32/100 vs 38.93/100, *P*<.001; and 35.71/100, *P*<.001) than during survey 2 and survey 4, respectively. This corresponds to pandemic peaks during which federal restrictions were better followed with self-reported adherence of 84.80/100 and 89.59/100 in survey 1 and survey 3 versus 78.69/100 (*P*<.001) and 78.64/100 (*P*<.001) in survey 2 and survey 4. A 2.9-point increase in worry score, adjusted for the pandemic period, gender, age, education, and health literacy, was associated with a 10-point increase in personal adherence score (95% CI 2.5-3.2; *P*<.001).

**Conclusions:**

Worries were higher in women, young people, and during the peak of the COVID-19 pandemic. Higher worry levels were associated with increased self-reported adherence to federal restrictions. Authorities should consider population worry levels and population subgroups in the planning and design of pandemic communication.

## Introduction

Effective communication between health authorities and the population is crucial to achieving public health goals during a pandemic. Providing clear, consistent, and reliable information that motivated behavior changes without triggering resistance was a major challenge during the COVID-19 pandemic. Sanitary restrictions were often rapidly issued and modified to contain the spread of the disease [1-3]. Citizens were expected to make drastic behavioral changes.

Public health authorities stressed the seriousness and risks of the pandemic to justify restrictions and encourage citizens’ adherence to them. In support of such an approach, the Health Belief Model argues that preventive health behaviors are influenced by perceived susceptibility to illness, the severity of the disease, benefits of and barriers to health-promoting actions, cues to action, as well as self-efficacy [4]. Also, during the COVID-19 pandemic, protective behaviors were associated with these factors, especially when the “perceived benefit” of a measure was clear [5].

Appealing to emotions such as fear can hence be a persuasive way of motivating respect for protective measures [6,7]. Indeed, fear leads to behavioral change if people feel capable of dealing with the threat, while they become defensive when feeling helpless and incapable of acting [8-10]. Overdriven or ill-conceived fear-based communication may even provoke counterproductive behavior.

Levels of anxiety, worry, and stress were high during the pandemic. According to a systematic review and meta-analysis, anxiety prevalence was around 30% worldwide after the first COVID-19 wave [11]. Others have confirmed these findings [12,13]. Among professionally active persons, 42% of participants reported being worried about the COVID-19 pandemic in August and September 2020 [14]. Young adults in the city of Zurich, Switzerland, reported elevated stress levels in April 2020, in the aftermath of the first wave [15], as was found more generally in Swiss adults too [16]. In terms of risk factors, anxiety was higher in women, younger people, and vulnerable persons [17-19].

In our previously published cross-sectional population survey, performed during the first wave of the pandemic, we found high self-reported adherence to official restrictions, which increased with age and level of worry [20]. As in the aforementioned studies, worry was high, particularly among people in isolation and with lower health literacy. Nearly half of the respondents felt that government responses were adequate or, associated with higher levels of worry, even insufficient. Neither the aforementioned nor our cross-sectional study could determine the evolution of these associations throughout the pandemic.

Thus, we conducted surveys during different phases of the pandemic to describe the evolution of worries in the Swiss population as well as associations between worries and adherence to governmental restrictions. Our overall aim was to contribute new insights to this understudied area to help improve crisis communication during future pandemics.

## Methods

### Study Design and Setting

We conducted repeated cross-sectional online surveys in Vaud, a French-speaking canton of 823,000 inhabitants (2021) in Switzerland. We launched 4 surveys between April 2020 and December 2021: survey 1 between April 17 and May 14, 2020 (4 weeks); survey 2 between May 15 and June 22, 2020 (5.5 weeks); survey 3 between October 30 and December 1, 2020 (4.5 weeks); and survey 4 between June 18 and December 30, 2021 (28 weeks, Table 1). Some of the survey items were adapted or replaced to capture changes in federal measures. We followed the CHERRIES (Checklist for Reporting Results of Internet e-Surveys) guidelines [21]. Self-reported worry was an outcome of the study of worry levels and an exposure variable for the study of associations between worry and self-reported adherence during these 4 COVID-19 pandemic periods.

**Table 1:** Surveyed periods and sentinel pandemic-related events.

Survey	Period	Number of weeks	Sentinel events
Survey 1: End of the first pandemic wave	April 17, 2020, to May 14, 2020	4	March 16, 2020: Semiconfinement, only essential shops open, gatherings of a maximum of 5 people April 27, 2020: Partial reopening of shops May 11, 2020: Reopening of schools
Survey 2: After the first pandemic wave	May 15, 2020, to June 22, 2020	5.5	June 19, 2020: End of an extraordinary situation
Survey 3: During the second pandemic wave	October 30, 2020, to December 1, 2020	4.5	Mandatory wearing of masks in indoor public spaces; gatherings limited to 15 people
Survey 4: Following pandemic waves	June 18, 2021, to December 30, 2021	28	Vaccination available to all, use of COVID-19 vaccination certificate

Table 1 presents the COVID-19 waves and their duration in the French-speaking part of Switzerland. The first and second forms were distributed at the end of the first wave, corresponding to the gradual emergence from confinement. The third form was distributed over a longer period, which included the second wave and the resumption of restrictive measures. The fourth form was distributed once vaccination was available for the entire population.

### Participant Recruitment

Using a weblink, we distributed the surveys on the social media platforms of multiple community organizations to collect a convenience sample of the population. These organizations were a regional consumer organization, regional disease leagues for cancer and diabetes, the association of senior citizens as well as the cantonal websites for the COVID-19 testing and vaccination decisions. These cantonal sites were used by large portions of the population. The organizations advertised the study through links on their websites and some social media accounts. The links were accompanied by a short explanation of the study and its purpose. No incentives to participate were given. The online interface for the survey was created in REDCap (Research Electronic Data Capture, Vanderbilt University).

### Surveys

The development and testing of the survey are described in our previous publication with results from survey 1 [20]. Each survey was submitted to 5 nonmedical persons to test the understandability of questions. There was no review step for this short questionnaire. The first, second, and fourth surveys had 20 items, and the third survey had 25 items. A total of 12 items remained unchanged throughout all surveys. We included demographic data, such as age, sex, number of persons per household, canton of residence, level of education, literacy, and whether the respondent had been tested for the COVID-19 pandemic. For the literacy question, we used a validated item from Chew et al [22]. Employment status was included in surveys 2-4. Respondents rated (1) worry about the pandemic situation, (2) self-reported adherence to government restrictions, and (3) perceived adherence of others to government restrictions, on visual analog scales from 0 to 100 (0=not at all; 100=in all situations). Items were not randomized. Participants could go back to earlier questions at any time. Adaptive questioning was used for several items. The first 2 surveys took up 6 screens, and the 2 latter, 7. REDCap automatically generated a completeness variable if participants went all the way to the end of the survey. Anyone who opened the survey generated a response. We did not determine unique site visitors, establish view or participation rates, or IP address checks, as surveys were entirely anonymous and the risk of repeating them was low. The time used to fill them in was not registered. No cookies were used, and there were no other techniques to analyze the log file of our database. Statistical corrections were not used. The 4 surveys can be found in Multimedia Appendices 1-4.

### Statistical Analyses

The item “Level of education” was dichotomized into “university or college education” or other. Health literacy was dichotomized according to ease of answering a medical form on one’s own: low literacy (“never,” “rarely,” or “sometimes” at ease) and “high literacy” (“often” or “always”). This was based on a validated item [22] and our previous article [20].

We limited our analysis to complete questionnaires for 2 reasons. First, the survey was distributed using an online link on government websites and a large number of persons clicked the link but only completed 1 or 2 questions. Second, no single question had many missing responses and we preferred to maintain consistency across surveys. We calculated means with SD and frequencies with IQR as appropriate. Independence between surveys was tested with the *t* test for continuous variables (eg, age), and with the chi-square test for gender, education, and health literacy. We performed linear regressions to analyze associations between the 4 periods and level of worry (model A), self-reported adherence (model B), and perceived adherence of others to restrictions (model C). First, we performed univariate linear regression, followed by multivariable linear regression controlling for age (grouped as 18-39 years, 40-64 years, and 65 years or older), sex (dichotomized male-female), level of education (dichotomized university education or other), and health literacy (dichotomized high or low health literacy). We subsequently used the margins command in Stata (StataCorp) to report absolute differences in the predicted levels of worry or adherence with each model. The level of significance was set to *P*<.05. Statistical analyses were performed in Microsoft Excel and Stata (version 16.1).

### Ethical Considerations

According to the Cantonal Commission on Ethics in Research Involving Human Beings of the Canton of Vaud, Switzerland the study was exempted from ethical review because it did not qualify as human subjects research and all data collection was anonymous (2024-010901).

## Results

### Participant Characteristics

**Figure 1:**
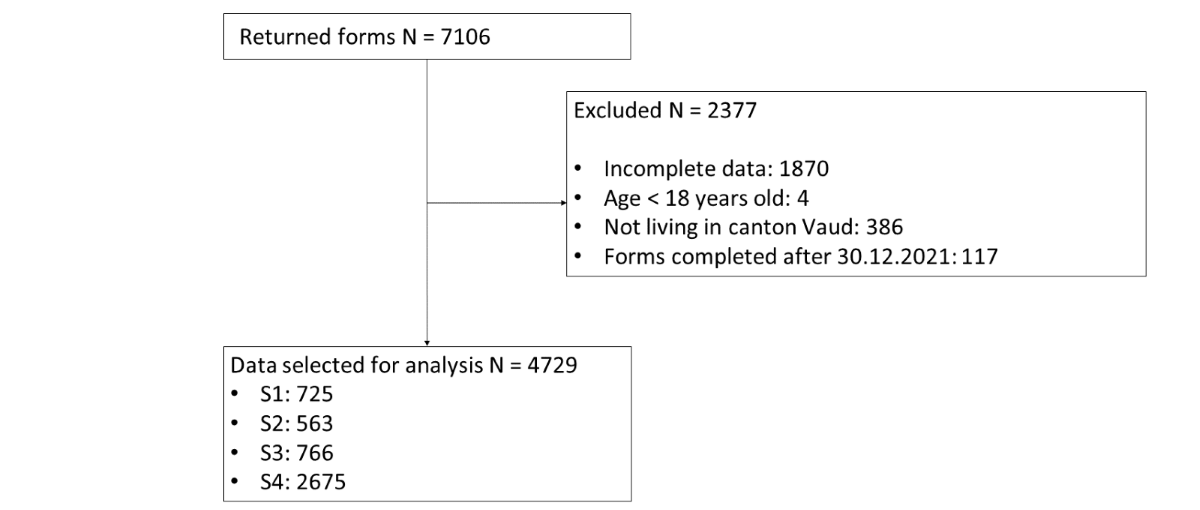
Study participants.

Citizens completed 7106 surveys between April 17, 2020, and December 20, 2021. After the exclusion of minors, persons living outside the Canton, and incomplete questionnaires, 4729 (66%) surveys remained. The number of questionnaires per period ranged between 563 and 2675 (Figure 1).

The participants were more often women (3025/4729, 63.96%) and between 40 and 64 years old (2442/4729, 51.64%). Furthermore, 2526/4729 (53.42%) of participants had attended university or college, and 4510/4729 (95.37%) reported high health literacy. The participants were younger in survey 3 (mean age 43.2, SD 14.6 years; *P*<.001) than in the other surveys (survey 1: mean age 47.9, SD 14.6 years; survey 2: mean age 47.3, SD 15.6 years; and survey 4: mean age 48.7, SD 15.3 years; Table 2).

**Table 2:** Demographics, education, health literacy, and employment of participants (n=4729).

Variables			All surveys		Survey 1^a^		Survey 2^b^		Survey 3^c^		Survey 4^d^
**Age (years), n (%)**											
	18-39	1574 (33.3)		244 (33.7)		202 (35.9)		325 (42.4)		803 (30)	
	40-64	2442 (51.6)		365 (50.3)		268 (47.6)		369 (48.2)		1440 (53.8)	
	≥65	713 (15.1)		116 (16)		93 (16.5)		72 (9.4)		432 (16.1)	
Years, mean (SD)			47.5 (15.3)		47.9 (14.6)		47.3 (15.6)		43.2 (14.6)		48.7 (15.3)
**Gender, n (%)**											
	Male	1698 (35.9)		168 (23.2)		178 (31.6)		264 (34.5)		1088 (40.7)	
	Female	3025 (64)		557 (76.8)		384 (68.2)		502 (65.5)		1582 (59.1)	
	Other^e^	6 (0.1)		0 (0)		1 (0.2)		0 (0)		5 (0.2)	
**Education, n (%)**											
	Obligatory school or less	256 (5.4)		12 (1.7)		21 (3.7)		28 (3.7)		195 (7.3)	
	Apprenticeship	1379 (29.2)		171 (23.6)		131 (23.3)		209 (27.3)		868 (32.4)	
	High-school graduation	502 (10.6)		69 (9.5)		47 (8.3)		85 (11.1)		301 (11.3)	
	University or college	2526 (53.4)		466 (64.3)		358 (63.6)		434 (56.7)		1268 (47.4)	
	I do not know	66 (1.4)		7 (1)		6 (1.1)		10 (1.3)		42 (1.6)	
**Health literacy, n (%)**											
	Low health literacy	219 (4.6)		52 (7.2)		33 (5.9)		74 (9.7)		344 (12.9)	
	High health literacy	4510 (95.4)		673 (92.8)		529 (94.1)		692 (90.3)		2326 (87.1)	
**Employment^f^, n (%)**											
	Full-time work	1707 (42.6)		—^g^		225 (40.0)		347 (45.3)		1135 (42.4)	
	Part-time work	674 (16.8)		—		102 (18.1)		136 (17.8)		436 (16.3)	
	Housewife and husband	125 (3.1)		—		18 (3.2)		24 (3.1)		83 (3.1)	
	Self-employed	303 (7.6)		—		37 (6.6)		46 (6.0)		220 (8.2)	
	Student	241 (6)		—		31 (5.5)		72 (9.4)		138 (5.2)	
**Employment status, n (%)**											
	Unemployed and currently looking for a job	133 (3.3)		—		23 (4.1)		19 (2.5)		91 (3.4)	
	Unemployed and not currently seeking employment	57 (1.4)		—		8 (1.4)		14 (1.8)		35 (1.3)	
	Incapacity	129 (3.2)		—		11 (2)		26 (3.4)		92 (3.4)	
	Retired	619 (15.5)		—		105 (18.7)		81 (10.6)		433 (16.2)	
	Unknown	15 (0.4)		—		3 (0.5)		1 (0.1)		11 (0.4)	

^a^Survey 1: April 17 to May 14, 2020.

^b^Survey 2: May 15 to June 22, 2020.

^c^Survey 3: October 30 to December 1, 2020.

^d^Survey 4: June 18, 2021, to December 30, 2021.

^e^These were excluded from the regression analyses.

^f^Employment data were not collected during the first survey.

^g^Not available.

### Main Results

#### Self-Reported Worry

**Figure 2:**
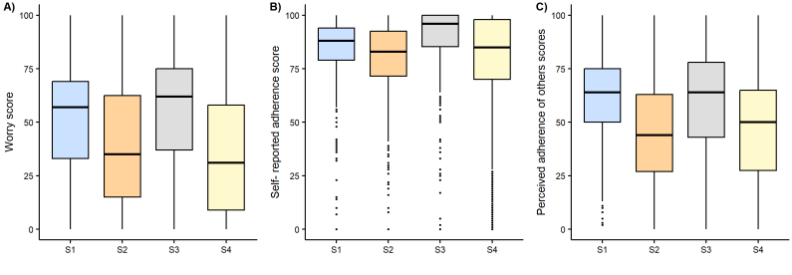
Boxplots on a 0-100 scale of (A) worry, (B) self-reported adherence, and (C) perceived adherence of others to restrictions during four COVID-19 pandemic periods (April 17, 2020, to December 30, 2021), Switzerland. S: survey; survey 1: April 17 to May 14, 2020; survey 2: May 15 to June 22, 2020; survey 3: October 30 to December 1, 2020; survey 4: June 18, 2021, to December 30, 2021.

Overall, the mean level of self-reported worry was 42.0% (SD 28.9). Upon univariate regression, self-reported worry levels differed significantly across surveys, with significantly higher levels in survey 1 (52%, 95% CI 50-54) and survey 3 (56%, 95% CI 54-58; Figure 2). Upon multivariable regression, the female gender was associated with a 4-point increase in level of worry (95% CI 2-5 points). Worry levels were 2 (95% CI 1-4) and 4 (95% CI 1-6) points lower among respondents aged 40 to 64 and over 64, respectively, when compared with the 18- to 39-year group. Higher health literacy was associated with a 3-point lower worry level (95% CI –6 to –1). Education was not associated with significant changes in self-reported level of worry (Table S5 in Multimedia Appendix 5).

#### Self-Reported Adherence to Restrictions

Overall, respondents evaluated their own adherence to government restrictions at 81.4% (SD 21.1). Self-reported adherence was significantly (*P*<.001) higher in survey 1 (mean 84.8%, SD 14.2) and survey 3 (mean 89.6%, SD 15.5; Figure 2). A 2.9-point increase in the worry score was associated with a 10-point increase in self-reported adherence (95% CI 2.5-3.2; *P*<.001) after adjusting for the pandemic period, gender, age, education, and health literacy. These effects were more pronounced in women and older participants (Table S5 in Multimedia Appendix 5). Moreover, both older age categories (40-64 years and more than 64 years) were associated with a 7-point higher self-reported adherence than in the 18-39 years group (95% CI 6-8 for 40- to 64-year-olds and 6-9 for >64-year-olds). Higher health literacy was associated with a 4-point increase in self-reported adherence (95% CI 2-6) while the educational level was not (Table S5 in Multimedia Appendix 5).

#### Perceived Adherence of Others to Restrictions

Overall, respondents evaluated the adherence of others to government restrictions at 50.4% (SD 24.5%). Evaluated adherence did not differ between survey 1 and survey 3 nor between survey 2 and survey 4 but was significantly higher (*P*<.001) in survey 1 (mean 60.1%, SD 20.0%) and survey 3 (59.6%, SD 24.6%) than in survey 2 (45.1%, SD 22.5%) and survey 4 (46.2%, SD 24.5%; Figure 2). Adjusting for participant characteristics, age groups 40-64 years and >64 years were associated with, respectively, a 5- or 7-point higher perceived adherence of others (95% CI 3-6 and 5-9, respectively). A 10-point increase in worry level was associated with a 1-point decrease in perceived adherence of others to restrictions (95% CI –0.9 to –0.4). Higher education level was associated with a 1.5-point higher perceived adherence of others (95% CI, 0-3), whereas gender and health literacy were not (Table S5 in Multimedia Appendix 5).

#### Changes to the Daily Lives of Respondents

During the first period (survey 1), most respondents had experienced changes in their daily life (Table S6 in Multimedia Appendix 6). Later (surveys 2-4), 35% to 49% had either lost their job or had to close their business and 21% to 34% had lost part of their income. Respondents also reported feeling isolated, lonelier, and less productive during surveys 2 to 4. In decreasing order of importance, concerns during survey 3 were for “vulnerable people,” “living conditions,” the “economy,” “self and family,” “working conditions,” and the “possibility of another wave.” During survey 4, these concerns were similar but generally rated lower. Interestingly, “deterioration of working conditions” moved up from fifth to third rank, which had been “self and family” in survey 3 (Multimedia Appendix Table S7 in Multimedia Appendix 7 and Table S8 in Multimedia Appendix 8).

## Discussion

### Principal Findings

We conducted online surveys during different phases of the COVID-19 pandemic to describe the evolution of worry levels and to assess how these were associated with adherence to government restrictions. In Switzerland, the self-reported worry was highest during the first and second pandemic waves, corresponding to survey 1 and survey 3, at times of many COVID-19–related hospitalizations and deaths, and when a vaccine was not yet available. Women and younger people reported higher levels of worry than men and older people. Education did not influence worry levels, while lower health literacy was associated with higher worry. Higher worry levels were associated with higher self-reported and perceived adherence of others to federal restrictions*.*

We found elevated worry levels during the more dramatic phases of the pandemic. In a systematic review covering 204 countries in 2020, higher anxiety was associated with higher COVID-19 incidence [23], a finding that was confirmed by Salanti et al [13]. In March 2020, Fitzpatrick et al [24] found that in a national sample in the United States, worry was higher in the regions with high COVID-19 incidence. In Ontario, Canada, COVID-19–related worry in young persons also increased during the early phases of the pandemic and then again in the autumn of 2020, when the incidence was higher [25]. So, anxiety and worry varied during the pandemic and increased repeatedly with the rising incidence of COVID-19. This is in line with findings on decreased mental health on a larger scale during the pandemic [11,26].

In periods of increased worry, we found higher self-reported adherence to government restrictions. Similarly, a study in Saudi Arabia described an association between higher anxiety levels and preventive practices among health care workers [27]. Another study identified fear as a predictor of behavioral change [28]. The association between worry and adherence in our study could indicate that worry was not overwhelming and that citizens felt in control of risks by respecting restrictions. We cannot exclude that this might have been different with higher anxiety levels.

Considering population subgroups, young adults were often the most anxious despite being less at risk of hospitalization or death [17,18,23,29]. Young people were worried about social isolation and develop depressive symptoms during school closings [30]. In Switzerland, students were concerned about whether they would be able to finish the 2020 university year [31], and lockdowns as well as their socioeconomic consequences were stressful for students [15]. Apart from concerns about the future, young people were not only worried about their own health but also about that of relatives. For example, in a study in Zurich, Switzerland, students were more concerned about the health of their parents and grandparents than their own [32]. Also, our finding of higher worry levels in women echoes several publications [17-19,33]. General factors potentially contributing to worry were the increasing risk of unemployment or loss of income, as well as loneliness and feeling less productive. One-third of respondents had lost part of their income by surveys 2 and 3 (31%, and 34%, respectively), with a slightly better situation in survey 4 (21%). The reported feelings of isolation, loneliness, and being less productive could further contribute to worry in some respondents. For example, studying for exams through online classes only, without any campus life, can be a source of worry compared with when stress from studying and exams is compensated by in-person interactions with teachers and colleagues. Four years after the pandemic, Sayed et al. [34] insist on the importance of addressing mental health of children and young adults during global crises and of recognizing long-term impacts. They further emphasize the need for research and public health prioritization of these important topics.

Overall, our findings are in line with publications that highlight the importance of addressing the many individual and collective aspects that influenced mental health during the COVID-19 pandemic, such as isolation, loneliness, and fear [35]. Many individuals demonstrated remarkable resilience, allowing society to avoid a general increase in loneliness [36]. However, population estimates may mask individual heterogeneity; loneliness is indeed a major public health concern and must be considered as a negative determinant of health [35]. Even though the pandemic is over, we must not forget its long-term effects on mental health and public health authorities should consider the differing impact of governmental decisions on the general population versus on individuals with pre-existing mental health conditions [37]. Our results of the worries of citizens and adherence to pandemic measures can be useful in preparing for future pandemics, for example, in considering criteria for and potential impact of restrictions on different subgroups of the population.

### Strengths and Limitations

The strengths of our study were repeated surveys with similar questions and our relatively large sample size, allowing us to examine subgroups of the population.

Concerning limitations, data were collected through the online distribution of surveys in a simple, cost-effective, and feasible way during the rapidly evolving pandemic. Participation was more attractive to women and younger people with higher literacy and education. Also, participation was variable during data collection periods. The participant sample was more representative in the last and longest period (survey 4). This selection and variation need to be considered in the interpretation of our data which are prone to desirability, information, and selection bias. Different distribution channels and methods are probably needed for disadvantaged populations, as we showed in a recent study using our survey in a population of refugees and migrants [38]. Finally, another inherent limitation of our anonymous data collection is that we could not follow a cohort of persons throughout the pandemic.

### Conclusion

Worry reached moderate levels and varied with COVID-19 incidence during the pandemic. Higher worry levels were associated with increased self-reported and perceived adherence of others to government restrictions. Younger people and women reported higher worry levels. Authorities should take population worry levels into account in planning and designing pandemic communication. Adapting communication to population subgroups should be considered for future health crises**.**

## Appendix

Multimedia Appendix 1 Survey 1.

Multimedia Appendix 2 Survey 2.

Multimedia Appendix 3 Survey 3.

Multimedia Appendix 4 Survey 4.

Multimedia Appendix 5 Linear regression results for three models; all on visual analogue scales from 0 to 100 (0=not at all; 100=in all situations).

Multimedia Appendix 6 Perceived changes of daily life during the first pandemic wave.

Multimedia Appendix 7 Impact of restrictions on daily life, S2, S3, S4.

Multimedia Appendix 8 Concerns of respondents, S3, S4.

## Abbreviations

CHERRIES: Checklist for Reporting Results of Internet e-Surveys
REDCap: Research Electronic Data Capture

## Data Availability

The datasets generated during and/or analyzed during this study are available from the corresponding author on reasonable request.
