# Supplementary material for: Citizen Worry and Adherence in Response to Government Restrictions in Switzerland During the COVID-19 Pandemic: Repeated Cross-Sectional Online Surveys
Source: Interact J Med Res. 2025 Jan 7;14:e55636. doi: 10.2196/55636 (PMC11751645; doi:10.2196/55636)
Supplement: Multimedia Appendix 1 [file ijmr_v14i1e55636_app1.pdf]

Code du dictionnaire de données

COVID-19 - Citizen (PID: 194)

09/06/2024 4:04pm

Formulaires

#	Nom de variable/champ	Étiquette de champ *Note de champ*	Attributs de champ (type de champ, validation, choix, logique de branchement, calculs, etc.)
Formulaire : **Reponses_citoyens_pandemie 4** (reponses_citoyens_pandemie_4) Enabled as survey [collapsed]			
Formulaire : **Reponses_citoyens_pandemie 3** (reponses_citoyens_pandemie_3) Enabled as survey [collapsed]			
Formulaire : **Reponses_citoyens_pandemie 2** (reponses_citoyens_pandemie_2) Enabled as survey [collapsed]			
Formulaire : **Reponses_citoyens_pandemie** (reponses_citoyens_pandemie) Enabled as survey			
101	[age_fr]	Quel âge avez-vous ?	text (integer, Min. : 18, Max. : 105), Required<br>Nombre de questions : 1
102	[gender_fr]	Quel est votre sexe?	radio, Required<br>1 Masculin<br>2 Féminin<br>3 Autre<br><br>Alignement personnalisé : RH<br>Nombre de questions : 2
103	[house_fr]	Combien de personnes, vous y compris, abrite votre domicile ?	text (integer, Min. : 1, Max. : 40)<br>Alignement personnalisé : RH<br>Nombre de questions : 3
104	[canton_fr]	Dans quel Canton vivez-vous ?	dropdown<br>22 Vaud(VD)<br>1 Zürich(ZH)<br>2 Aargau(AG)<br>3 Appenzell(Appenzell Ausserrhoden(AR) /Appenzell Innerrhoden(AI))<br>4 Basel(Basel Stadt(BS)/Basel Land(BL))<br>5 Bern / Berne(BE)<br>6 Freiburg / Fribourg(FR)<br>7 Genève(GE)<br>8 Glarus(GL)<br>9 Graubünden(GR)<br>10 Jura(JU)

			11 Luzern(LU)<br>12 Neuchâtel(NE)<br>13 Sankt Gallen(SG)<br>14 Schaffhausen(SH)<br>15 Schwyz(SZ)<br>16 Solothurn(SO)<br>17 Thurgau(TG)<br>18 Ticino(TI)<br>19 Unterwalden(Obwalden(OW) /Nidwalden(NW))<br>20 Uri(UR)<br>21 Valais / Wallis(VS)<br>23 Zug(ZG)<br><br>Alignement personnalisé : RH<br>Nombre de questions : 4
105	[education_fr]	Quel est votre plus haut niveau de formation ?	radio<br>1 Ecole obligatoire ou moins<br>2 Apprentissage<br>3 Maturité gymnasiale<br>4 Haute école ou université<br>5 Je ne sais pas<br><br>Nombre de questions : 5
106	[literacy_fr]	Êtes-vous à l'aise pour remplir vous-même un formulaire médical ? (p. ex. questionnaire nouveau patient chez un nouveau médecin)	radio, Required<br>1 Jamais<br>2 Rarement<br>3 Parfois<br>4 Souvent<br>5 Toujours<br><br>Nombre de questions : 6
107	[test_fr]	Avez-vous été testé.e pour le nouveau coronavirus (Covid-19) ?	radio, Required<br>1 Oui, testé.e positif.ve<br>2 Oui, testé.e négatif.ve<br>3 Oui, en attente du résultat<br>4 Non<br>5 Je ne sais pas<br><br>Nombre de questions : 7

<table>
<tr><td>108</td><td>[symptom_fr]</td><td>Avez-vous actuellement les symptômes suivants ? (Veuillez sélectionner tous les symptômes présents)</td><td>checkbox, Required<table><tr><td>1</td><td>symptom_fr___1</td><td>Fièvre (≥38°C, sensation fébrile, frissons)</td></tr><tr><td>2</td><td>symptom_fr___2</td><td>Toux</td></tr><tr><td>3</td><td>symptom_fr___3</td><td>Peine à respirer</td></tr><tr><td>4</td><td>symptom_fr___4</td><td>Autres:</td></tr><tr><td>5</td><td>symptom_fr___5</td><td>Aucun, je me sens bien</td></tr><tr><td>6</td><td>symptom_fr___6</td><td>Je ne sais pas</td></tr></table>Nombre de questions : 8</td></tr>
<tr><td>109</td><td>[symptom_other_fr]
Afficher le champ U NIQUEMENT si : [symptom_fr(4)] = '1'</td><td>Veuillez préciser</td><td>text, Required
Alignement personnalisé : LH
Annotation de champ: @WORDLIMIT 50</td></tr>
<tr><td>110</td><td>[info_fr]</td><td>En-tête de section : Information concernant le nouveau coronavirus (SARS CoV-19)
Quelles sont vos trois sources principales d'information pour rester à jour concernant les recommandations pour le nouveau coronavirus (Covid-19) ? (3 réponses maximum)
Limit 3 responses</td><td>checkbox, Required<table><tr><td>1</td><td>info_fr___1</td><td>Les sites web et annonces du gouvernement</td></tr><tr><td>2</td><td>info_fr___2</td><td>Informations dans les journaux ou la presse électronique</td></tr><tr><td>3</td><td>info_fr___3</td><td>Les informations transmises par télévision ou radio</td></tr><tr><td>4</td><td>info_fr___4</td><td>Blogs et autres sources d'information non formelles</td></tr><tr><td>5</td><td>info_fr___5</td><td>Réseaux sociaux (p. ex. Facebook, Instagram, Whatsapp, Twitter, ...)</td></tr><tr><td>6</td><td>info_fr___6</td><td>Votre employeur.euse</td></tr><tr><td>7</td><td>info_fr___7</td><td>Votre famille et vos ami.e.s</td></tr><tr><td>8</td><td>info_fr___8</td><td>Les hotlines téléphoniques du gouvernement</td></tr><tr><td>9</td><td>info_fr___9</td><td>Votre médecin ou le site internet d'un hôpital ou clinique</td></tr></table></td></tr>
</table>

			10 | info_fr___10 | Je ne reste pas à jour concernant le nouveau coronavirus (COVID-19)<br>11 | info_fr___11 | Autres :<br><br>Alignement personnalisé : LV<br>Nombre de questions : 9<br>Annotation de champ: @MAXCHECKED=3
111	[info_other_fr]<br><br>Afficher le champ U NIQUEMENT si :<br>[info_fr(11)] = '1'	Veuillez préciser	text, Required<br>Alignement personnalisé : LH<br>Annotation de champ: @WORDLIMIT 50
112	[worry_fr]	De manière générale, à quel point êtes-vous inquiet.ète concernant le nouveau coronavirus (Covid-19)	slider (Min. : 0, Max. : 100), Required<br>Étiquettes de défilement : pas du tout inquiet.ète, , très inquiet.ète<br>Alignement personnalisé : LH<br>Nombre de questions : 10
113	[knowledge_fr]	Parmi les propositions suivantes, quelles sont les recommandations actuelles des autorités pour diminuer la propagation du nouveau coronavirus (Covid-19) ?<br>(Sélectionner toutes les réponses possibles)	checkbox<br>1 | knowledge_fr___1 | Se laver les mains régulièrement durant 20 secondes<br>2 | knowledge_fr___2 | Passer le plus de temps possible à l'extérieur chaque jour<br>3 | knowledge_fr___3 | Laver soigneusement tous les fruits et légumes<br>4 | knowledge_fr___4 | Se tenir à une distance de 2 mètres des autres personnes<br>5 | knowledge_fr___5 | En cas de fièvre ou toux, rester à domicile pendant 10 jours et 48 heures après la disparition des symptômes<br>6 | knowledge_fr___6 | Arrêter d'utiliser les

			| | | transports publics | | 7 | knowledge_fr___7 | Annuler tous les évènements publics | | 8 | knowledge_fr___8 | Eviter les mélanges inter-générationnels dans la famille (p. ex. les grands-parents qui s'occupent des enfants) | | 9 | knowledge_fr___9 | Faire autant que possible du télétravail | <br>Alignement personnalisé : LV<br>Nombre de questions : 11
114	[changes_fr]	En-tête de section : *Apporter des changements à votre routine dû au nouveau coronavirus (COVID-19)*<br><br>Avez-vous été confronté.e à une ou plusieurs mesures spécifiques ci-dessous en raison des restrictions liées au nouveau coronavirus (Covid-19) ? (Sélectionner toutes les réponses possibles)	checkbox, Required<br>| 1 | changes_fr___1 | Annulation de voyages organisés | | 2 | changes_fr___2 | Annulation d'évènements culturels, sportifs, ... | | 3 | changes_fr___3 | Jours de travail manqués | | 4 | changes_fr___4 | Télétravail | | 5 | changes_fr___5 | Eviter le contact physique avec d'autres personnes (p.ex. éviter les poignées de main) | | 6 | changes_fr___6 | Annulation d'engagement dans le cadre d'activités sociales | | 7 | changes_fr___7 | Mise en auto-quarantaine ou auto-isolement, ne pas sortir du domicile durant plusieurs jours consécutifs | | 8 | changes_fr___8 | Aucune mesure |

			9 | changes_fr___9 | Autres changements :<br><br>Alignement personnalisé : LV<br>Nombre de questions : 12
115	[changes_other_fr]<br><br>Afficher le champ UNIQUEMENT si :<br>[changes_fr(9)] = '1'	Veuillez préciser	text, Required<br>Alignement personnalisé : LH<br>Annotation de champ: @WORDLIMIT=50
116	[restrictions_fr_v3]	A l'heure actuelle, avez-vous le sentiment que les recommandations des autorités pour limiter la propagation du nouveau coronavirus (Covid-19) ont été :	slider (Min. : 0, Max. : 100), Required<br>Étiquettes de défilement : pas de tout suffisantes, suffisantes, beaucoup trop restrictives<br>Alignement personnalisé : LH<br>Nombre de questions : 19
117	[protect_fr]	Quelles mesures avez-vous mis en place pour protéger votre famille et vos ami.e.s proches ? (Sélectionner toutes les réponses possibles)	checkbox, Required<br>1 | protect_fr___1 | Je leur ai transmis des informations<br>2 | protect_fr___2 | Je les ai aidé.e.s à limiter leur exposition à de nombreuses personnes<br>3 | protect_fr___3 | J'ai évité de les rencontrer<br>4 | protect_fr___4 | Je les ai évité.e.s lorsque j'avais des symptômes (p. ex. toux, fièvre, ...)<br>5 | protect_fr___5 | Je leur ai fourni de la nourriture ou d'autres essentiels<br>6 | protect_fr___6 | Cette question ne s'applique pas à ma situation<br>7 | protect_fr___7 | Autres :<br><br>Nombre de questions : 13
118	[protect_other_fr]<br><br>Afficher le champ UNIQUEMENT si :<br>[protect_fr(7)] = '1'	Veuillez préciser:	text, Required<br>Alignement personnalisé : LH<br>Annotation de champ: @WORDLIMIT = 50
119	[help_fr]	Qu'est-ce qui vous a aidé à mettre en place ces mesures spécifiques ? (Sélectionner toutes les réponses possibles)	checkbox, Required<br>1 | help_fr___1 | Aide de la famille ou des ami.e.s<br>2 | help_fr___2 | Aide de l'employeur.euse

			3 | help_fr___3 | Remboursement de frais de la part d'une assurance ou autre (p. ex. remboursement de réservation de voyage)<br>4 | help_fr___4 | Informations reçues sur internet<br>5 | help_fr___5 | Informations reçues de la part de la famille ou des ami.e.s<br>6 | help_fr___6 | Informations reçues de la part du gouvernement<br>7 | help_fr___7 | En appliquant des conseils ou astuces pour rendre les recommandations plus faciles à appliquer<br>8 | help_fr___8 | En suivant l'exemple d'autres personnes<br>9 | help_fr___9 | Je ne suis pas les recommandations<br>10 | help_fr___10 | Autres :<br><br>Alignement personnalisé : LV<br>Nombre de questions : 14
120	[help_other_fr]<br>Afficher le champ UNIQUEMENT si :<br>[help_fr(10)] = '1'	Veuillez préciser:	text, Required<br>Alignement personnalisé : LH<br>Annotation de champ:<br>@WORDLIMIT=50
121	[barrier_fr]	Qu'est-ce qui vous a empêché, même occasionnellement, de mettre en place ces mesures spécifiques ? (Sélectionner toutes les réponses possibles)	checkbox, Required<br>1 | barrier_fr___1 | Je ne pense pas que ces mesures soient utiles pour prévenir une contamination<br>2 | barrier_fr___2 | Je ne pense pas que le virus soit dangereux pour moi-même ou pour ma famille<br>3 | barrier_fr___3 | C'est difficile de changer mes habitudes

<table>
<tr><td></td><td></td><td></td><td>
<table>
<tr><td>4</td><td>barrier_fr___4</td><td>Je ne veux pas offenser les autres (p. ex. de ne pas serrer la main à quelqu'un qui me la tend)</td></tr>
<tr><td>5</td><td>barrier_fr___5</td><td>Je m'inquiète de manquer des jours de travail ou de décevoir mon employeur</td></tr>
<tr><td>6</td><td>barrier_fr___6</td><td>Obligation de quitter ma maison pour des besoins essentiels (p. ex. faire des courses)</td></tr>
<tr><td>7</td><td>barrier_fr___7</td><td>Obligations familiales (p.ex. la nécessité de m'occuper d'enfants ou de personnes âgées)</td></tr>
<tr><td>8</td><td>barrier_fr___8</td><td>Je m'inquiète de mon état de santé (p. ex. la nécessité d'une consultation chez le médecin)</td></tr>
<tr><td>9</td><td>barrier_fr___9</td><td>Je ne me rappelle pas ou ne comprends pas les recommandations</td></tr>
<tr><td>10</td><td>barrier_fr___10</td><td>Je n'ai pas les moyens de mettre en place ces mesures (p. ex. vie en collocation)</td></tr>
<tr><td>11</td><td>barrier_fr___11</td><td>Je suis systématiquement les conseils sans problèmes</td></tr>
<tr><td>12</td><td>barrier_fr___12</td><td>Autres :</td></tr>
</table>
Alignement personnalisé : LV
Nombre de questions : 15
</td></tr>
<tr><td>122</td><td>[barrier_other_fr]

Afficher le champ UNIQUEMENT si :
[barrier_fr(12)] = '1'</td><td>Veuillez préciser</td><td>text, Required
Alignement personnalisé : LH
Annotation de champ: @WORDLIMIT = 50</td></tr>
<tr><td>123</td><td>[adhere_fr]</td><td>En-tête de section : Suivre les recommandations gouvernementales</td><td>slider (Min. : 0, Max. : 100), Required
Étiquettes de défilement : pas du tout, , en toute situation</td></tr>
</table>

		De manière générale, à quel point avez-vous, vous-même, suivi les recommandations fédérales, telle que se laver les mains, se tenir à une distance de 2 mètres des autres personnes, ne pas toucher le visage, ... ?	Alignement personnalisé : LH<br>Nombre de questions : 16
124	[adhere_2_fr]	De manière générale, à quel point avez-vous constaté que les autres personnes ont suivi les recommandations fédérales, telle que se laver les mains, se tenir à une distance de 2 mètres des autres personnes, ne pas toucher le visage, ... ?	slider (Min. : 0, Max. : 100), Required<br>Étiquettes de défilement : pas du tout, , en toute situation<br>Alignement personnalisé : LH<br>Nombre de questions : 17
125	[intervene_fr]	Êtes-vous intervenu afin d'encourager d'autres personnes à suivre les recommandations ?	radio, Required<br>1 Oui<br>2 Non<br>3 Je ne sais pas<br><br>Nombre de questions : 18
126	[restrictions_fr]	A l'heure actuelle, avez-vous le sentiment que les recommandations des autorités pour limiter la propagation du nouveau coronavirus (Covid-19) ont été :	slider (Min. : 0, Max. : 100), Required<br>Étiquettes de défilement : pas de tout suffisantes, suffisantes, beaucoup trop restrictives<br>Alignement personnalisé : LH<br>Nombre de questions : 19
127	[ideas_fr]	A votre avis, que pourraient faire les autorités afin d'aider les citoyen.ne.s à limiter la propagation du nouveau coronavirus (Covid-19) ?	notes<br>Alignement personnalisé : LV<br>Nombre de questions : 20<br>Annotation de champ: @WORDLIMIT = 200
128	[reponses_citoyens_pandemie_complete]	En-tête de section : *Form Status*<br>Complete?	dropdown<br>0 Incomplete<br>1 Unverified<br>2 Complete

Formulaire : Citizen Responses To The Covid19 Pandemic (citizen_responses_to_the_covid19_pandemic) **Enabled as survey** **[collapsed]**
