# Supplementary material for: Citizen Worry and Adherence in Response to Government Restrictions in Switzerland During the COVID-19 Pandemic: Repeated Cross-Sectional Online Surveys
Source: Interact J Med Res. 2025 Jan 7;14:e55636. doi: 10.2196/55636 (PMC11751645; doi:10.2196/55636)
Supplement: Multimedia Appendix 2 [file ijmr_v14i1e55636_app2.pdf]

Code du dictionnaire de données

COVID-19 - Citizen (PID: 194)

09/06/2024 4:04pm

Formulaires

#	Nom de variable/champ	Étiquette de champ<br>*Note de champ*	Attributs de champ (type de champ, validation, choix, logique de branchement, calculs, etc.)
Formulaire : **Reponses_citoyens_pandemie 4** (reponses_citoyens_pandemie_4) Enabled as survey [collapsed]			
Formulaire : **Reponses_citoyens_pandemie 3** (reponses_citoyens_pandemie_3) Enabled as survey [collapsed]			
Formulaire : **Reponses_citoyens_pandemie 2** (reponses_citoyens_pandemie_2) Enabled as survey			
72	[age_v2]	Quel âge avez-vous ?	text (integer, Min. : 18, Max. : 105), Required<br>Nombre de questions : 1
73	[gender_v2]	Quel est votre sexe ?	radio, Required<br>1 Masculin<br>2 Féminin<br>3 Autre<br><br>Alignement personnalisé : RH<br>Nombre de questions : 2
74	[canton_v2]	Dans quel canton vivez-vous ?	dropdown, Required<br>22 Vaud(VD)<br>1 Zürich(ZH)<br>2 Aargau(AG)<br>3 Appenzell(Appenzell Ausserrhoden(AR) /Appenzell Innerrhoden(AI))<br>4 Basel(Basel Stadt(BS)/Basel Land(BL))<br>5 Bern / Berne(BE)<br>6 Freiburg / Fribourg(FR)<br>7 Genève(GE)<br>8 Glarus(GL)<br>9 Graubünden(GR)<br>10 Jura(JU)<br>11 Luzern(LU)<br>12 Neuchâtel(NE)<br>13 Sankt Gallen(SG)<br>14 Schaffhausen(SH)<br>15 Schwyz(SZ)

			16 Solothurn(SO)<br>17 Thurgau(TG)<br>18 Ticino(TI)<br>19 Unterwalden(Obwalden(OW) /Nidwalden(NW))<br>20 Uri(UR)<br>21 Valais / Wallis(VS)<br>23 Zug(ZG)<br><br>Alignement personnalisé : RH<br>Nombre de questions : 3
75	[education_v2]	Quel est votre plus haut niveau de formation ?	radio, Required<br>1 Ecole obligatoire ou moins<br>2 Apprentissage<br>3 Maturité gymnasiale<br>4 Haute école ou université<br>5 Je ne sais pas<br><br>Nombre de questions : 4
76	[employment_v2]	Quelle est votre situation professionnelle actuelle ?	radio, Required<br>1 Employé.e à temps plein (32 heures ou plus par semaine)<br>2 Employé.e à temps partiel (moins de 32 heures par semaine)<br>3 Femme/homme au foyer<br>4 Indépendant.e<br>5 Étudiant.e<br>6 Sans emploi et actuellement à la recherche d'un emploi<br>7 Sans emploi et ne cherche pas actuellement un emploi<br>8 Incapacité de travail<br>9 Retraité.e<br>10 Je ne sais pas<br><br>Nombre de questions : 5
77	[literacy_v2]	Êtes-vous à l'aise pour remplir vous-même un formulaire médical ? (p. ex. questionnaire nouveau patient chez un nouveau médecin)	radio, Required<br>1 Jamais<br>2 Rarement<br>3 Parfois<br>4 Souvent<br>5 Toujours

			Nombre de questions : 6
78	[test_v2]	Avez-vous été testé.e pour le nouveau coronavirus (Covid-19) ?	radio, Required<br>1 Oui, testé.e positif.ve<br>2 Oui, testé.e négatif.ve<br>3 Oui, en attente du résultat<br>4 Non<br>5 Je ne sais pas<br><br>Nombre de questions : 7
79	[symptom_v2]	Avez-vous actuellement les symptômes suivants ? (Veuillez sélectionner tous les symptômes présents)	checkbox, Required<br>1 symptom_v2___1 Fièvre (≥38°C, sensation fébrile, frissons)<br>2 symptom_v2___2 Toux<br>3 symptom_v2___3 Peine à respirer<br>4 symptom_v2___4 Autres:<br>5 symptom_v2___5 Aucun, je me sens bien<br>6 symptom_v2___6 Je ne sais pas<br><br>Nombre de questions : 8
80	[symptom_other_v2]<br>Afficher le champ UNIQUEMENT si : [symptom_v2(4)] = '1'	Veuillez préciser	text, Required<br>Alignement personnalisé : LH<br>Annotation de champ: @WORDLIMIT 50
81	[risk_v2]	Faites-vous partie d'un groupe à risque de Covid-19 ? Cela signifie que vous avez été diagnostiqué ou êtes traité pour de l'hypertension, du diabète, une maladie cardiaque (p. ex: crise cardiaque précédente), maladie pulmonaire chronique (p. ex: asthme ou bronchite chronique), ou vous-êtes immunodéprimé en raison d'une maladie ou d'un traitement.	radio, Required<br>1 Oui<br>2 Non<br>3 Je ne sais pas<br><br>Nombre de questions : 9
82	[worry_v2]	En-tête de section : *Information concernant le nouveau coronavirus (Covid-19)*<br>Au cours des 5 derniers jours, à quel point êtes-vous inquiet.ète à propos du nouveau coronavirus (Covid-19) ?	slider (Min. : 0, Max. : 100), Required<br>Étiquettes de défilement : pas du tout inquiet.ète, , très inquiet.ète<br>Alignement personnalisé : LH
83	[knowledge_v2]	Parmi les propositions suivantes, quelles sont les recommandations actuelles des autorités pour diminuer la propagation du nouveau coronavirus (Covid-19) ? (Sélectionner toutes les réponses possibles)	checkbox<br>1 knowledge_v2___1 Passer le plus de temps possible à l'extérieur chaque jour

			2 | knowledge_v2___2 | En cas de fièvre ou de toux, rester à domicile pendant 10 jours et 48 heures après la disparition symptômes<br>3 | knowledge_v2___3 | Arrêter d'utiliser les transports publics<br>4 | knowledge_v2___4 | Se tenir à une distance de 2 mètres des autres personnes<br>5 | knowledge_v2___5 | Les rassemblements de 5 à 15 personnes sont désormais autorisés<br>6 | knowledge_v2___6 | Faire autant que possible du télétravail<br>7 | knowledge_v2___7 | Les magasins vendant des vêtements sont maintenant ouverts<br>8 | knowledge_v2___8 | Les restaurants, bars et pubs sont ouverts à tous, à condition que les clients restent debout ou dehors sur une terrasse<br><br>Alignement personnalisé : LV<br>Nombre de questions : 11
84	[impact_v2]	En-tête de section : *Changements dans votre vie dû au nouveau coronavirus (Covid-19)*<br><br>Quel impact les restrictions du coronavirus (Covid-19) ont-elles eu sur votre vie ? (Sélectionner toutes les réponses possibles)	checkbox, Required<br>1 | impact_v2___1 | J'ai perdu mon emploi ou j'ai dû fermer mon entreprise en raison des restrictions<br>2 | impact_v2___2 | J'ai conservé mon emploi, mais j'ai perdu mon revenu en raison

			(suite) d'heures de travail manquées<br>3 impact_v2___3 J'ai été moins productif.ve car j'ai dû changer mes habitudes de travail<br>4 impact_v2___4 J'ai été plus seul.e parce que je ne vois ni famille ni amis<br>5 impact_v2___5 J'ai été plus anxieux.se à cause des changements dans ma routine<br>6 impact_v2___6 Je me suis isolé.e ou mis.e en auto-quarantaine, je n'ai pas quitté mon domicile pendant plusieurs jours consécutifs<br>7 impact_v2___7 Les restrictions n'ont pas eu d'impact significatif sur ma vie<br>8 impact_v2___8 Autre :<br>Alignement personnalisé : LV<br>Nombre de questions : 12
85	[impact_other_v2]<br>Afficher le champ UNIQUEMENT si : [impact_v2(8)] = '1'	Veuillez préciser	text, Required<br>Alignement personnalisé : LH<br>Annotation de champ: @WORDLIMIT=50
86	[easier_v2]	Après deux mois de pandémie de Covid-19, trouvez-vous que les restrictions gouvernementales sont plus faciles ou plus difficiles à suivre qu'en début de pandémie ?	slider (Min. : 0, Max. : 100), Required<br>Étiquettes de défilement : plus facile avec le temps, pas de changement, plus difficile avec le temps<br>Alignement personnalisé : RH<br>Nombre de questions : 13
87	[maintenir_v2]	Quelles recommandations ont été difficiles à maintenir au fil du temps ? (Sélectionner toutes les réponses possibles)	checkbox, Required<br>1 maintenir_v2___1 Maintien de l'éloignement physique des autres (ex: 2 mètres dans les magasins)<br>2 maintenir_v2___2 Éviter le mélange intergénérationnel (ex: ne pas voir

			<table><tr><td></td><td></td><td>ses parents ou ses grands-parents)</td></tr><tr><td>3</td><td>maintenir_v2___3</td><td>Ne pas voir ses amis.es</td></tr><tr><td>4</td><td>maintenir_v2___4</td><td>Ne pas aller dans les magasins ou utiliser les services normalement disponibles</td></tr><tr><td>5</td><td>maintenir_v2___5</td><td>Eviter les regroupements de plus de 5 personnes</td></tr><tr><td>6</td><td>maintenir_v2___6</td><td>Auto-isolement en cas de symptômes ou auto-quarantaine</td></tr><tr><td>7</td><td>maintenir_v2___7</td><td>Autre:</td></tr></table><br>Alignement personnalisé : LV<br>Nombre de questions : 14
88	[maintenir_other_v2]<br><br>Afficher le champ UNIQUEMENT si :<br>[maintenir_v2(7)] = '1'	Veuillez préciser	text, Required<br>Alignement personnalisé : LH<br>Annotation de champ: @WORDLIMIT = 50
89	[adhere_v2]	En-tête de section : *Suivre les recommandations gouvernementales*<br><br>Au cours des 5 derniers jours, à quel point avez-vous, vous-même, suivi les recommandations fédérales, telle que se laver les mains, se tenir à une distance de 2 mètres des autres personnes, ne pas toucher le visage, ... ?	slider (Min. : 0, Max. : 100), Required<br>Étiquettes de défilement : pas du tout, , en toute situation<br>Alignement personnalisé : LH<br>Nombre de questions : 15
90	[adhere_2_v2]	Au cours des 5 derniers jours, à quel point avez-vous constaté que les autres personnes ont suivi les recommandations fédérales, telle que se laver les mains, se tenir à une distance de 2 mètres des autres personnes, ne pas toucher le visage, ... ?	slider (Min. : 0, Max. : 100), Required<br>Étiquettes de défilement : pas du tout, , en toute situation<br>Alignement personnalisé : LH<br>Nombre de questions : 16
91	[lever_v2]	Pensez-vous que les changements mis en œuvre le 27 avril et 11 mai pour commencer à lever les restrictions liées au coronavirus (COVID-19) étaient :	slider (Min. : 0, Max. : 100), Required<br>Étiquettes de défilement : trop permissifs, appropriés, trop restrictifs<br>Alignement personnalisé : LH<br>Nombre de questions : 17
92	[restrictions_v4]	Avez-vous le sentiment que les recommandations des autorités pour limiter la propagation du nouveau coronavirus (Covid-19) jusqu' à ce jour ont été :	slider (Min. : 0, Max. : 100), Required<br>Étiquettes de défilement : pas de tout suffisantes, suffisantes, beaucoup trop restrictives<br>Alignement personnalisé : LH<br>Nombre de questions : 18

93	[tracing_v2]	La confédération va bientôt tester une nouvelle application sur téléphone mobile. Cette application permet de savoir, sans révéler l'identité des utilisateurs, si vous avez côtoyé une personne testée positive depuis. Accepteriez-vous d'utiliser cette application sur votre téléphone?	radio, Required<br>1 Oui<br>2 Non<br><br>Nombre de questions : 19
94	[tracing_yes_v2]<br><br>Afficher le champ UNIQUEMENT si :<br>[tracing_v2] = '1'	Si oui, sous quelles conditions ?	text, Required
95	[contact_v2]	Quelles mesures de suivi et de transmission de votre localisation et de votre état de santé trouvez-vous acceptables :	checkbox<br>1 contact_v2___1 Partager mes données avec les responsables de la santé publique<br>2 contact_v2___2 Être informé par les responsables de la santé publique que j'ai été en contact avec une personne qui a le Covid-19<br>3 contact_v2___3 Permettre aux restaurants, magasins ou autres établissements publiques de me contacter si j'ai été en contact avec une personne qui a le Covid-19<br>4 contact_v2___4 Permettre à mon employeur d'informer mes collègues si je développe le Covid-19<br>5 contact_v2___5 Permettre aux responsables de la santé publique d'informer mes voisins si j'ai le Covid-19<br>6 contact_v2___6 Permettre aux responsables de la santé publique de vérifier que je suis resté en auto-isolement

			| | | pendant 10 jours si j'ai le Covid-19 | | 7 | contact_v2___7 | Autre: | <br>Annotation de champ: @HIDDEN-SURVEY
96	[contact_other_v2]	Veuillez préciser	text, Required<br>Alignement personnalisé : LH<br>Annotation de champ: @HIDDEN-SURVEY
97	[contact_no_v2]<br><br>Afficher le champ UNIQUEMENT si : [tracing_v2] = '2'	Pourquoi n'accepteriez-vous d'utiliser une telle application sur votre téléphone ? (Sélectionner toutes les réponses possibles)	checkbox, Required<br>| 1 | contact_no_v2___1 | Cela envahie sur ma vie privée | | 2 | contact_no_v2___2 | Si le gouvernement obtient ces droits maintenant, ils perdureront après la crise | | 3 | contact_no_v2___3 | Les dangers posés par Covid-19 ne justifient pas des telles mesures | | 4 | contact_no_v2___4 | Je suis confiant.e que les recommandation déjà en place son suffisantes | | 5 | contact_no_v2___5 | Si je suis infecté.e par le Covid-19, je ne veux pas partager cette information | | 6 | contact_no_v2___6 | Je ne fais pas confiance au gouvernement | | 7 | contact_no_v2___7 | Autre: |
98	[tracing_other_v2]<br><br>Afficher le champ UNIQUEMENT si : [contact_no_v2(7)] = '1'	Veuillez préciser	text, Required<br>Alignement personnalisé : LH<br>Annotation de champ: @WORDLIMIT = 50
99	[ideas_v2]	Quelles autres mesures pourrait prendre le gouvernement pour aider les citoyens à limiter la propagation du coronavirus (COVID-19) au moment où de nouvelles directives sont mises en œuvre (à partir du 27 avril et 11 mai)?	notes<br>Alignement personnalisé : LV<br>Nombre de questions : 20<br>Annotation de champ: @WORDLIMIT = 200
100	[reponses_citoyens_pandemie_2_complete]	En-tête de section : *Form Status*<br><br>Complete?	dropdown<br>| 0 | Incomplete |

1	Unverified
2	Complete

Formulaire : **Reponses_citoyens_pandemie** (reponses_citoyens_pandemie) Enabled as survey [collapsed]

Formulaire : **Citizen Responses To The Covid19 Pandemic** (citizen_responses_to_the_covid19_pandemic) Enabled as survey [collapsed]
