# Supplementary material for: Citizen Worry and Adherence in Response to Government Restrictions in Switzerland During the COVID-19 Pandemic: Repeated Cross-Sectional Online Surveys
Source: Interact J Med Res. 2025 Jan 7;14:e55636. doi: 10.2196/55636 (PMC11751645; doi:10.2196/55636)
Supplement: Multimedia Appendix 3 [file ijmr_v14i1e55636_app3.pdf]

Code du dictionnaire de données

COVID-19 - Citizen (PID: 194)

09/06/2024 4:04pm

Formulaires

#	Nom de variable/champ	Étiquette de champ<br>*Note de champ*	Attributs de champ (type de champ, validation, choix, logique de branchement, calculs, etc.)
Formulaire : **Reponses_citoyens_pandemie 4** (reponses_citoyens_pandemie_4) Enabled as survey [collapsed]			
Formulaire : **Reponses_citoyens_pandemie 3** (reponses_citoyens_pandemie_3) Enabled as survey			
36	[age_v3]	Quel âge avez-vous ?	text (integer, Min. : 18, Max. : 105), Required
37	[gender_v3]	Quel est votre sexe ?	radio, Required<br>1 Masculin<br>2 Féminin<br>3 Autre<br><br>Alignement personnalisé : RH
38	[canton_v3]	Dans quel canton vivez-vous ?	dropdown, Required<br>22 Vaud(VD)<br>1 Zürich(ZH)<br>2 Aargau(AG)<br>3 Appenzell(Appenzell Ausserrhoden(AR) /Appenzell Innerrhoden(AI))<br>4 Basel(Basel Stadt(BS)/Basel Land(BL))<br>5 Bern / Berne(BE)<br>6 Freiburg / Fribourg(FR)<br>7 Genève(GE)<br>8 Glarus(GL)<br>9 Graubünden(GR)<br>10 Jura(JU)<br>11 Luzern(LU)<br>12 Neuchâtel(NE)<br>13 Sankt Gallen(SG)<br>14 Schaffhausen(SH)<br>15 Schwyz(SZ)<br>16 Solothurn(SO)<br>17 Thurgau(TG)<br>18 Ticino(TI)

			19 Unterwalden(Obwalden(OW) /Nidwalden(NW))<br>20 Uri(UR)<br>21 Valais / Wallis(VS)<br>23 Zug(ZG)<br><br>Alignement personnalisé : RH
39	[education_v3]	Quel est votre plus haut niveau de formation ?	radio, Required<br>1 École obligatoire ou moins<br>2 Apprentissage<br>3 Maturité gymnasiale<br>4 Haute école ou université<br>5 Je ne sais pas
40	[employment_v3]	Quelle est votre situation professionnelle actuelle ?	radio, Required<br>1 Employé.e à temps plein (32 heures ou plus par semaine)<br>2 Employé.e à temps partiel (moins de 32 heures par semaine)<br>3 Femme/homme au foyer<br>4 Indépendant.e<br>5 Étudiant.e<br>6 Sans emploi et actuellement à la recherche d'un emploi<br>7 Sans emploi et ne cherche pas actuellement un emploi<br>8 Incapacité de travail<br>9 Retraité.e<br>10 Je ne sais pas
41	[literacy_v3]	Êtes-vous à l'aise pour remplir vous-même un formulaire médical ? (p. ex. questionnaire nouveau patient chez un nouveau médecin)	radio, Required<br>1 Jamais<br>2 Rarement<br>3 Parfois<br>4 Souvent<br>5 Toujours
42	[test_v3]	Avez-vous été testé.e une fois ou plus pour le nouveau coronavirus (Covid-19) ?	radio, Required<br>1 Oui, avec au moins un test positif<br>2 Oui, avec toujours des résultats négatifs<br>3 Oui, en attente du résultat<br>4 Non<br>5 Je ne sais pas

<table>
<tr><td>43</td><td>[worry_v3]</td><td>En-tête de section : Information concernant le nouveau coronavirus (Covid-19)

Au cours des 5 derniers jours, à quel point êtes-vous inquiet.ète à propos du nouveau coronavirus (Covid-19) ?</td><td>slider (Min. : 0, Max. : 100), Required
Étiquettes de défilement : pas du tout inquiet.ète, , très inquiet.ète
Alignement personnalisé : LH</td></tr>
<tr><td>44</td><td>[knowledge_v3]</td><td>Parmi les propositions suivantes, quelles sont les recommandations actuelles des autorités pour diminuer la propagation du nouveau coronavirus (Covid-19) ? (Sélectionner toutes les réponses possibles)</td><td>checkbox
<table>
<tr><td>1</td><td>knowledge_v3___1</td><td>Passer le plus de temps possible à l'extérieur chaque jour</td></tr>
<tr><td>2</td><td>knowledge_v3___2</td><td>Se tenir à une distance de 1.5 mètres des autres personnes</td></tr>
<tr><td>3</td><td>knowledge_v3___3</td><td>En cas de test Covid-19 positif, rester à domicile pendant 10 jours et 48 heures après la disparition des symptômes</td></tr>
<tr><td>4</td><td>knowledge_v3___4</td><td>Arrêter d'utiliser les transports publics</td></tr>
<tr><td>5</td><td>knowledge_v3___5</td><td>Faire autant que possible du télétravail, si votre employeur/activité le permet</td></tr>
<tr><td>6</td><td>knowledge_v3___6</td><td>Il faut rester à domicile pendant 2 jours en revenant d'un pays à risque</td></tr>
<tr><td>7</td><td>knowledge_v3___7</td><td>Le port du masque est recommandé dans tous les espaces publiques</td></tr>
<tr><td>8</td><td>knowledge_v3___8</td><td>Les restaurants et bars sont ouverts mais seulement le service aux tables est permis</td></tr>
</table>
Alignement personnalisé : LV</td></tr>
<tr><td>45</td><td>[impact_v3]</td><td>Quel impact les restrictions du coronavirus (Covid-19) ont-elles sur votre vie en ce moment? (Sélectionner toutes les réponses possibles)</td><td>checkbox, Required
<table>
<tr><td>1</td><td>impact_v3___1</td><td>J'ai perdu mon emploi ou j'ai dû fermer mon entreprise</td></tr>
<tr><td>2</td><td>impact_v3___2</td><td>J'ai perdu une partie de mon</td></tr>
</table></td></tr>
</table>

			revenu (chômage partiel, diminution de mon taux d'activité,...)<br>3 impact_v3___3 Je me sens moins productif.ve<br>4 impact_v3___4 Je me sens plus seul.e<br>5 impact_v3___5 Je me sens isolé.e<br>6 impact_v3___6 Les restrictions n'ont pas d'impact significatif sur ma vie<br>7 impact_v3___7 Autre:<br><br>Alignement personnalisé : LV
46	[impact_other_v3]<br><br>Afficher le champ UNIQUEMENT si :<br>[impact_v3(7)] = '1'	Veuillez préciser	text, Required<br>Alignement personnalisé : LH<br>Annotation de champ: @WORDLIMIT = 50
47	[vulnerable_v3]	En-tête de section : *Quelle est votre attitude face à la pandémie COVID-19 ?*<br><br>Êtes-vous préoccupé-e pour les personnes les plus vulnérables ?	radio (Matrice), Required<br>1 Pas du tout d'accord<br>2 Plutôt pas d'accord<br>3 Ni d'accord, ni pas d'accord<br>4 Plutôt d'accord<br>5 Tout à fait d'accord
48	[economy_v3]	Êtes-vous préoccupé-e pour l'économie ?	radio (Matrice), Required<br>1 Pas du tout d'accord<br>2 Plutôt pas d'accord<br>3 Ni d'accord, ni pas d'accord<br>4 Plutôt d'accord<br>5 Tout à fait d'accord
49	[work_v3]	Êtes-vous préoccupé-e par la détérioration des conditions de travail ?	radio (Matrice), Required<br>1 Pas du tout d'accord<br>2 Plutôt pas d'accord<br>3 Ni d'accord, ni pas d'accord<br>4 Plutôt d'accord<br>5 Tout à fait d'accord
50	[life_v3]	Êtes-vous préoccupé-e par la détérioration des conditions de vie ?	radio (Matrice), Required<br>1 Pas du tout d'accord<br>2 Plutôt pas d'accord<br>3 Ni d'accord, ni pas d'accord

			4 Plutôt d'accord<br>5 Tout à fait d'accord
51	[family_v3]	Êtes-vous préoccupé-e pour vous ou pour votre famille ?	radio (Matrice), Required<br>1 Pas du tout d'accord<br>2 Plutôt pas d'accord<br>3 Ni d'accord, ni pas d'accord<br>4 Plutôt d'accord<br>5 Tout à fait d'accord
52	[depressed_v3]	Êtes-vous déprimé-e par cette deuxième vague ?	radio (Matrice), Required<br>1 Pas du tout d'accord<br>2 Plutôt pas d'accord<br>3 Ni d'accord, ni pas d'accord<br>4 Plutôt d'accord<br>5 Tout à fait d'accord
53	[adhere_v3]	Au cours des 5 derniers jours, à quel point avez-vous, vous-même, suivi les recommandations concernant les gestes-barrière (tels que se laver ou se désinfecter les mains, se tenir à une distance de 1.5 m des autres personnes, ne pas se toucher le visage, ...) lorsque vous avez rencontré des proches, collègues ou amis ?	slider (Min. : 0, Max. : 100), Required<br>Étiquettes de défilement : pas du tout, , en toute situation<br>Alignement personnalisé : LH
54	[adhere_v4]	Au cours des 5 derniers jours, à quel point avez-vous, vous même, suivi les recommandations concernant les gestes-barrière (tels que se laver ou se désinfecter les mains, se tenir à une distance de 1.5 m des autres personnes, ne pas se toucher le visage, ...) dans les lieux publics au contact d'inconnus ?	slider (Min. : 0, Max. : 100), Required<br>Étiquettes de défilement : pas du tout, , en toute situation<br>Alignement personnalisé : LH
55	[adhere2_v4]	Au cours des 5 derniers jours, à quel point avez-vous constaté que les autres personnes ont suivi les recommandations concernant les gestes-barrière (tels que se laver ou se désinfecter les mains, se tenir à une distance de 1.5 m des autres personnes, ne pas se toucher le visage, ...) durant des rencontres avec des proches, collègues ou amis ?	slider (Min. : 0, Max. : 100), Required<br>Étiquettes de défilement : pas du tout, , en toute situation<br>Alignement personnalisé : LH
56	[adhere2_v3]	Au cours des 5 derniers jours, à quel point avez-vous constaté que les autres personnes ont suivi les recommandations concernant les gestes-barrière (tels que se laver les mains, se tenir à une distance de 1.5 mètres des autres personnes, ne pas se toucher le visage, ...) dans les lieux publics ?	slider (Min. : 0, Max. : 100), Required<br>Étiquettes de défilement : pas du tout, , en toute situation<br>Alignement personnalisé : LH
57	[restrictions_v3]	En-tête de section : *Votre opinion des recommandations gouvernementales*<br>Avez-vous le sentiment que les recommandations des autorités pour limiter	slider (Min. : 0, Max. : 100), Required<br>Étiquettes de défilement : pas de tout suffisantes, suffisantes, beaucoup trop restrictives<br>Alignement personnalisé : LH

		la propagation du nouveau coronavirus (Covid-19) sont :	
58	[ideas_v3]	Quelles autres mesures pourrait prendre le gouvernement pour aider les citoyens à limiter la propagation du coronavirus (COVID-19) au moment où de nouvelles directives sont mises en œuvre (à partir du 19 et 28 octobre)?	notes<br>Alignement personnalisé : LV<br>Annotation de champ: @WORDLIMIT = 200
59	[tracingapp_v3]	Avez-vous téléchargé l'application SwissCovid ? (décrit dans cette vidéo youtube)	radio, Required<br>1 Oui<br>2 Non<br>3 Je ne sais pas
60	[quarantine_v3]	Selon vous, est-il toujours approprié de placer les contacts des personnes testées positives au Covid-19 en quarantaine ?	radio<br>1 Oui<br>2 Non<br>3 Je ne sais pas
61	[quarantine_no_v3]<br>Afficher le champ U NIQUEMENT si : [quarantine_v3] = '2'	Si non, pensez vous que :	checkbox, Required<br>1 quarantine_no_v3___1 La quarantaine est inefficace<br>2 quarantine_no_v3___2 La quarantaine est difficile à appliquer<br>3 quarantine_no_v3___3 La longueur de la quarantaine est excessive<br>4 quarantine_no_v3___4 L'impact économique est trop important<br>5 quarantine_no_v3___5 Autre
62	[quarantine_no_other]<br>Afficher le champ U NIQUEMENT si : [quarantine_no_v3(5)] = '1'	Veuillez préciser	text, Required<br>Alignement personnalisé : LH<br>Annotation de champ: @WORDLIMIT = 50
63	[vaccine_v3]	Lorsque le vaccin contre le coronavirus sera disponible, vous ferez-vous vacciner ?	radio, Required<br>1 Oui, le plus rapidement possible<br>2 Oui, après quelque mois, le temps d'avoir un peu de recul sur les effets<br>3 Non<br>4 Je ne sais pas

64	[vaccine_yes_v3]<br><br>Afficher le champ UNIQUEMENT si :<br>[vaccine_v3] = '1' or [vaccine_v3] = '2'	Si oui, quelles sont vos motivations pour vous faire vacciner ? (plusieurs réponses possibles)	checkbox, Required<br>1 vaccine_yes_v3___1 Me protéger du virus<br>2 vaccine_yes_v3___2 Protéger mes proches, amis et collègues du virus<br>3 vaccine_yes_v3___3 Protéger les personnes les plus vulnérables du virus<br>4 vaccine_yes_v3___4 Diminuer le risque d'une nouvelle épidémie<br>5 vaccine_yes_v3___5 Autre :
65	[vaccine_yes_other]<br><br>Afficher le champ UNIQUEMENT si :<br>[vaccine_yes_v3(5)] = '1'	Veuillez préciser	text, Required<br>Alignement personnalisé : LH<br>Annotation de champ: @WORDLIMIT = 50
66	[vaccine_no_v3]<br><br>Afficher le champ UNIQUEMENT si :<br>[vaccine_v3] = '3'	Si non, qu'est-ce qui vous retiens de vous faire vacciner ?	text, Required<br>Alignement personnalisé : LH<br>Annotation de champ: @WORDLIMIT = 200
67	[incentive_v3]<br><br>Afficher le champ UNIQUEMENT si :<br>[vaccine_v3] = '4' or [vaccine_v3] = '3'	Qu'est-ce qui vous encouragerait à vous faire vacciner ? (plusieurs réponses possibles)	checkbox<br>1 incentive_v3___1 Des informations scientifiques compréhensibles sur les risques et bénéfices<br>2 incentive_v3___2 Les recommandations des autorités publiques<br>3 incentive_v3___3 Les recommandations de votre médecin de confiance<br>4 incentive_v3___4 Je peux me faire vacciner au travail ou dans des lieux publics, pas seulement chez mon médecin de confiance ou dans des policliniques<br>5 incentive_v3___5 Le vaccin est gratuit

			6 | incentive_v3___6 | Le vaccin est remboursé par mon assurance maladie<br>7 | incentive_v3___7 | Dans aucun cas est-ce que je pense me faire vacciner<br>8 | incentive_v3___8 | Autre :<br><br>Alignement personnalisé : LV
68	[vaccine_yes_other_2]<br><br>Afficher le champ UNIQUEMENT si : [incentive_v3(8)] = '1'	Veuillez préciser	text, Required<br>Alignement personnalisé : LH<br>Annotation de champ: @WORDLIMIT = 50
69	[incentive_v4]<br><br>Afficher le champ UNIQUEMENT si : [vaccine_v3] = '2'	Qu'est-ce qui vous encouragerait à vous faire vacciner plus rapidement ? (plusieurs réponses possibles)	checkbox<br>1 | incentive_v4___1 | Des informations scientifiques compréhensibles sur les risques et bénéfices<br>2 | incentive_v4___2 | Les recommandations des autorités publiques<br>3 | incentive_v4___3 | Les recommandations de votre médecin de confiance<br>4 | incentive_v4___4 | Je peux me faire vacciner au travail ou dans des lieux publics, pas seulement chez mon médecin de confiance ou dans des policliniques<br>5 | incentive_v4___5 | Le vaccin est gratuit<br>6 | incentive_v4___6 | Le vaccin est remboursé par mon assurance maladie<br>7 | incentive_v4___7 | Je ne souhaite en aucun cas me faire vacciner<br>8 | incentive_v4___8 | Autre :<br><br>Alignement personnalisé : LV
70	[incentive_other_v3]	Veuillez préciser	text, Required<br>Alignement personnalisé : LH

	Afficher le champ UNIQUEMENT si : [incentive_v4(8)] = '1'		Annotation de champ: @WORDLIMIT = 50
71	[reponses_citoyens_pandemie_3_complete]	En-tête de section : *Form Status*<br>Complete?	dropdown<br>0 Incomplete<br>1 Unverified<br>2 Complete
Formulaire : **Reponses_citoyens_pandemie 2** (reponses_citoyens_pandemie_2) Enabled as survey [collapsed]			
Formulaire : **Reponses_citoyens_pandemie** (reponses_citoyens_pandemie) Enabled as survey [collapsed]			
Formulaire : **Citizen Responses To The Covid19 Pandemic** (citizen_responses_to_the_covid19_pandemic) Enabled as survey [collapsed]			
