# Supplementary material for: Citizen Worry and Adherence in Response to Government Restrictions in Switzerland During the COVID-19 Pandemic: Repeated Cross-Sectional Online Surveys
Source: Interact J Med Res. 2025 Jan 7;14:e55636. doi: 10.2196/55636 (PMC11751645; doi:10.2196/55636)
Supplement: Multimedia Appendix 4 [file ijmr_v14i1e55636_app4.pdf]

Code du dictionnaire de données

COVID-19 - Citizen (PID: 194)

09/06/2024 4:04pm

Formulaires

#	Nom de variable/champ	Étiquette de champ *Note de champ*	Attributs de champ (type de champ, validation, choix, logique de branchement, calculs, etc.)
**Formulaire : Reponses_citoyens_pandemie 4 (reponses_citoyens_pandemie_4) Enabled as survey**			
1	[record_id]	Record ID	text, Identifier
2	[age_v4]	Quel âge avez-vous ?	text (integer, Min. : 18, Max. : 105), Required
3	[gender_v4]	Quel est votre sexe ?	radio, Required<br>1 Masculin<br>2 Féminin<br>3 Autre<br><br>Alignement personnalisé : RH
4	[canton_v4]	Dans quel canton vivez-vous ?	dropdown, Required<br>22 Vaud(VD)<br>1 Zürich(ZH)<br>2 Aargau(AG)<br>3 Appenzell(Appenzell Ausserrhoden(AR) /Appenzell Innerrhoden(AI))<br>4 Basel(Basel Stadt(BS)/Basel Land(BL))<br>5 Bern / Berne(BE)<br>6 Freiburg / Fribourg(FR)<br>7 Genève(GE)<br>8 Glarus(GL)<br>9 Graubünden(GR)<br>10 Jura(JU)<br>11 Luzern(LU)<br>12 Neuchâtel(NE)<br>13 Sankt Gallen(SG)<br>14 Schaffhausen(SH)<br>15 Schwyz(SZ)<br>16 Solothurn(SO)<br>17 Thurgau(TG)<br>18 Ticino(TI)

			19 Unterwalden(Obwalden(OW) /Nidwalden(NW))<br>20 Uri(UR)<br>21 Valais / Wallis(VS)<br>23 Zug(ZG)<br><br>Alignement personnalisé : RH
5	[education_v4]	Quel est votre plus haut niveau de formation ?	radio, Required<br>1 École obligatoire ou moins<br>2 Apprentissage<br>3 Maturité gymnasiale<br>4 Haute école ou université<br>5 Je ne sais pas
6	[employment_v4]	Quelle est votre situation professionnelle actuelle ?	radio, Required<br>1 Employé.e à temps plein (32 heures ou plus par semaine)<br>2 Employé.e à temps partiel (moins de 32 heures par semaine)<br>3 Femme/homme au foyer<br>4 Indépendant.e<br>5 Étudiant.e<br>6 Sans emploi et actuellement à la recherche d'un emploi<br>7 Sans emploi et ne cherche pas actuellement un emploi<br>8 Incapacité de travail<br>9 Retraité.e<br>10 Je ne sais pas
7	[literacy_v4]	Êtes-vous à l'aise pour remplir vous-même un formulaire médical ? (p. ex. questionnaire nouveau patient chez un nouveau médecin)	radio, Required<br>1 Jamais<br>2 Rarement<br>3 Parfois<br>4 Souvent<br>5 Toujours
8	[test_v4]	Avez-vous été testé.e une fois ou plus pour le nouveau coronavirus (COVID-19) ?	radio, Required<br>1 Oui, avec au moins un test positif<br>2 Oui, avec toujours des résultats négatifs<br>3 Oui, en attente du résultat<br>4 Non

			5 Je ne sais pas
9	[worry_v4]	En-tête de section : *Information concernant le nouveau coronavirus (COVID-19)*<br>Au cours des 5 derniers jours, à quel point êtes-vous inquiet.ète à propos du nouveau coronavirus (COVID-19) ?	slider (Min. : 0, Max. : 100), Required<br>Étiquettes de défilement : pas du tout inquiet.ète, , très inquiet.ète<br>Alignement personnalisé : LH
10	[impact_v4]	Quel impact les restrictions du coronavirus (COVID-19) ont-elles sur votre vie en ce moment? (Sélectionner toutes les réponses possibles)	checkbox, Required<br>1 impact_v4___1 J'ai perdu mon emploi ou j'ai dû fermer mon entreprise<br>2 impact_v4___2 J'ai perdu une partie de mon revenu (chômage partiel, diminution de mon taux d'activité,...)<br>3 impact_v4___3 Je me sens moins productif.ve<br>4 impact_v4___4 Je me sens plus seul.e<br>5 impact_v4___5 Je me sens isolé.e<br>6 impact_v4___6 Les restrictions n'ont pas d'impact significatif sur ma vie en ce moment<br>7 impact_v4___7 Autre:<br>Alignement personnalisé : LV
11	[impact_other_v4]<br>Afficher le champ UNIQUEMENT si : [impact_v4(7)] = '1'	Veuillez préciser	text, Required<br>Alignement personnalisé : LH<br>Annotation de champ: @WORDLIMIT = 50
12	[vulnerable_v4]	En-tête de section : *Quelle est votre attitude face à la pandémie COVID-19 ?*<br>Êtes-vous préoccupé-e pour les personnes les plus vulnérables ?	radio (Matrice), Required<br>1 Pas du tout d'accord<br>2 Plutôt pas d'accord<br>3 Ni d'accord, ni pas d'accord<br>4 Plutôt d'accord<br>5 Tout à fait d'accord
13	[economy_v4]	Êtes-vous préoccupé-e pour l'économie ?	radio (Matrice), Required<br>1 Pas du tout d'accord<br>2 Plutôt pas d'accord<br>3 Ni d'accord, ni pas d'accord

			4 Plutôt d'accord<br>5 Tout à fait d'accord
14	[work_v4]	Êtes-vous préoccupé-e par la détérioration des conditions de travail ?	radio (Matrice), Required<br>1 Pas du tout d'accord<br>2 Plutôt pas d'accord<br>3 Ni d'accord, ni pas d'accord<br>4 Plutôt d'accord<br>5 Tout à fait d'accord
15	[life_v4]	Êtes-vous préoccupé-e par la détérioration des conditions de vie ?	radio (Matrice), Required<br>1 Pas du tout d'accord<br>2 Plutôt pas d'accord<br>3 Ni d'accord, ni pas d'accord<br>4 Plutôt d'accord<br>5 Tout à fait d'accord
16	[family_v4]	Êtes-vous préoccupé-e pour vous ou pour votre famille ?	radio (Matrice), Required<br>1 Pas du tout d'accord<br>2 Plutôt pas d'accord<br>3 Ni d'accord, ni pas d'accord<br>4 Plutôt d'accord<br>5 Tout à fait d'accord
17	[depressed_v4]	Êtes-vous déprimé-e par la possibilité d'une autre vague ?	radio (Matrice), Required<br>1 Pas du tout d'accord<br>2 Plutôt pas d'accord<br>3 Ni d'accord, ni pas d'accord<br>4 Plutôt d'accord<br>5 Tout à fait d'accord
18	[adhereout_v4]	Au cours des 5 derniers jours, à quel point avez-vous, vous même, suivi les recommandations concernant les gestes-barrière (tels que se laver ou se désinfecter les mains, se tenir à une distance de 1.5 m des autres personnes, ne pas se toucher le visage, ...) dans les lieux publics au contact d'inconnus ?	slider (Min. : 0, Max. : 100), Required<br>Étiquettes de défilement : pas du tout, , en toute situation<br>Alignement personnalisé : LH
19	[adhere2out_v4]	Au cours des 5 derniers jours, à quel point avez-vous constaté que les autres personnes ont suivi les recommandations concernant les gestes-barrière (tels que se laver les mains, se tenir à une distance de 1.5 mètres des autres personnes, ne pas se toucher le visage, ...) dans les lieux publics ?	slider (Min. : 0, Max. : 100), Required<br>Étiquettes de défilement : pas du tout, , en toute situation<br>Alignement personnalisé : LH
20	[vaccine_v4]	En-tête de section : *Vaccination COVID-19*	radio, Required

		Est-ce que vous êtes déjà vacciné.e contre le COVID-19? Avez-vous l'intention de vous faire vacciner?	1 Je suis vacciné.e avec au moins une dose<br>2 J'ai rendez-vous pour me faire vacciner<br>3 J'espère prendre rendez-vous pour me faire vacciner<br>4 Je reste en attente d'information ou indécis.e<br>5 Je ne veux pas me faire vacciner<br>6 Je ne sais pas
21	[vaccinated_v4]<br>Afficher le champ UNIQUEMENT si : [vaccine_v4] = '1'	Quelles étaient vos motivations pour vous faire vacciner ? (plusieurs réponses possibles)	checkbox, Required<br>1 vaccinated_v4___1 Me protéger du virus<br>2 vaccinated_v4___2 Protéger mes proches, amis et collègues du virus<br>3 vaccinated_v4___3 Protéger les personnes les plus vulnérables du virus<br>4 vaccinated_v4___4 Diminuer le risque d'une nouvelle épidémie<br>5 vaccinated_v4___5 Autre :
22	[vaccinated_other_v4]<br>Afficher le champ UNIQUEMENT si : [vaccinated_v4(5)] = '1'	Veuillez préciser	text, Required<br>Alignement personnalisé : LH<br>Annotation de champ: @WORDLIMIT = 50
23	[vaccine_yes_v4]<br>Afficher le champ UNIQUEMENT si : [vaccine_v4] = '2' or [vaccine_v4] = '3'	Si oui, quelles sont vos motivations pour vous faire vacciner ? (plusieurs réponses possibles)	checkbox, Required<br>1 vaccine_yes_v4___1 Me protéger du virus<br>2 vaccine_yes_v4___2 Protéger mes proches, amis et collègues du virus<br>3 vaccine_yes_v4___3 Protéger les personnes les plus vulnérables du virus

			4 vaccine_yes_v4___4 Diminuer le risque d'une nouvelle épidémie<br>5 vaccine_yes_v4___5 Autre :
24	[vaccine_yes_other_v4]<br><br>Afficher le champ UNIQUEMENT si : [vaccine_yes_v4(5)] = '1'	Veuillez préciser	text, Required<br>Alignement personnalisé : LH<br>Annotation de champ: @WORDLIMIT = 50
25	[vaccine_no_v4]<br><br>Afficher le champ UNIQUEMENT si : [vaccine_v4] = '5'	Si non, qu'est-ce qui vous retiens de vous faire vacciner ?	text, Required<br>Alignement personnalisé : LH<br>Annotation de champ: @WORDLIMIT = 200
26	[alternative_v4]<br><br>Afficher le champ UNIQUEMENT si : [vaccine_v4] = '5'	La prochaine question est ouverte, c'est vous qui choisissez la réponse, si vous souhaitez y répondre. Que voyez-vous comme approche alternative à la vaccination pour sortir de l'épidémie ?	notes
27	[incentive_v5]<br><br>Afficher le champ UNIQUEMENT si : [vaccine_v4] = '4'	Si vous n'avez pas encore décidé, qu'est-ce qui vous encouragerait à vous faire vacciner ? (plusieurs réponses possibles)	checkbox<br>1 incentive_v5___1 Des informations scientifiques compréhensibles sur les risques et bénéfices<br>2 incentive_v5___2 Il devient plus facile d'obtenir un rendez-vous pour se faire vacciner<br>3 incentive_v5___3 Les recommandations de votre médecin de confiance<br>4 incentive_v5___4 Si plus de personnes dans mon entourage se font vacciner sans complications<br>5 incentive_v5___5 Plus de recul, comme peut-être dans 6 mois ou l'année prochaine<br>6 incentive_v5___6 Si le vaccin devient obligatoire pour voyager<br>7 incentive_v5___7 Si le vaccin devient obligatoire pour les évènements

			sportifs et/ou culturels<br>8 incentive_v5___8 Dans aucun cas est-ce que je pense me faire vacciner<br>9 incentive_v5___9 Autre :<br><br>Alignement personnalisé : LV
28	[incentive_other_v5]<br><br>Afficher le champ UNIQUEMENT si :<br>[incentive_v5(9)] = '1'	Veuillez préciser	text, Required<br>Alignement personnalisé : LH<br>Annotation de champ: @WORDLIMIT = 50
29	[passport_v4]	On parle de la mise en place d'un document attestant d'une vaccination ou d'avoir fait la maladie (Certificat immunitaire, Certificat COVID-19...). Etes-vous favorable à un tel document ?	radio, Required<br>1 Tout à fait favorable<br>2 Favorable<br>3 Ni favorable/ni pas favorable<br>4 Pas favorable<br>5 Pas du favorable
30	[campaign_info_en_ligne]	Connaissez-vous la campagne d'information sur la vaccination > ?	radio<br>1 Oui<br>2 Non<br>3 Je ne sais pas<br><br>Alignement personnalisé : LV
31	[restrictions_gov_v4]	En-tête de section : *Votre opinion des recommandations gouvernementales*<br><br>Avez-vous le sentiment que les recommandations des autorités pour limiter la propagation du nouveau coronavirus (Covid-19) sont :	slider (Min. : 0, Max. : 100), Required<br>Étiquettes de défilement : pas de tout suffisantes, suffisantes, beaucoup trop restrictives<br>Alignement personnalisé : LH
32	[assouplissement_v4]	Le conseil fédéral a de nouveau assoupli les restrictions liées au COVID-19 depuis le 31 mai. Quelle est votre perception de ces changements :	radio<br>1 C'est beaucoup trop tôt<br>2 C'est un peu trop tôt<br>3 C'est le bon moment<br>4 Ca prend trop de temps<br>5 Ca prend beaucoup trop de temps<br><br>Alignement personnalisé : LV
33	[ideas_v4]	Quelles autres mesures pourrait prendre le gouvernement actuellement pour aider les citoyens à limiter la propagation du coronavirus (COVID-19) ?	notes<br>Alignement personnalisé : LV<br>Annotation de champ: @WORDLIMIT = 200

34	[knowledge_v4]	Parmi les propositions suivantes, quelles sont les recommandations actuelles des autorités pour diminuer la propagation du nouveau coronavirus (Covid-19) ?<br>(Sélectionner toutes les réponses possibles)	radio<br>1 Passer le plus de temps possible à l'extérieur chaque jour<br>2 Se tenir à une distance de 1.5 mètres des autres personnes<br>3 En cas de test Covid-19 positif, rester à domicile pendant 10 jours et 48 heures après la disparition des symptômes<br>4 Arrêter d'utiliser les transports publics<br>5 Faire autant que possible du télétravail, si votre employeur/activité le permet<br>6 Il faut rester à domicile pendant 2 jours en revenant d'un pays à risque<br>7 Le port du masque est recommandé dans tous les espaces publiques<br>8 Les restaurants et bars sont ouverts mais seulement le service aux tables est permis<br><br>Annotation de champ: @HIDDEN-SURVEY
35	[reponses_citoyens_pandemie_4_complete]	En-tête de section : *Form Status*<br>Complete?	dropdown<br>0 Incomplete<br>1 Unverified<br>2 Complete

Formulaire : **Reponses_citoyens_pandemie 3** (reponses_citoyens_pandemie_3) Enabled as survey [collapsed]

Formulaire : **Reponses_citoyens_pandemie 2** (reponses_citoyens_pandemie_2) Enabled as survey [collapsed]

Formulaire : **Reponses_citoyens_pandemie** (reponses_citoyens_pandemie) Enabled as survey [collapsed]

Formulaire : **Citizen Responses To The Covid19 Pandemic** (citizen_responses_to_the_covid19_pandemic) Enabled as survey [collapsed]
