# Supplementary material for: Citizen Worry and Adherence in Response to Government Restrictions in Switzerland During the COVID-19 Pandemic: Repeated Cross-Sectional Online Surveys
Source: Interact J Med Res. 2025 Jan 7;14:e55636. doi: 10.2196/55636 (PMC11751645; doi:10.2196/55636)
Supplement: Multimedia Appendix 5 [file ijmr_v14i1e55636_app5.pdf]

Supplementary table 5: Linear regression results for three models; all on visual analogue scales from 0 to 100 (0=not at all; 100=in all situations)

Model A: Worry about the COVID-19 pandemic situation

	**Coefficient**	***P-value***	**95%CI**
10-point increase<br>**Self-reported adherence**	2.87	<**0.001**	2.49 to 3.25
**Period**			
S1	Ref		
S2	-11.4	<**0.001**	-14.4 to -8.4
S3	2.53	0.072	-0.23 to 5.29
S4	-14.4	<**0.001**	-16.7 to -12.2
**Age**			
18-39	Ref		
40-64	-2.50	**0.005**	-4.24 to -0.76
>64	-3.87	**0.002**	-6.31 to -1.44
**Gender**			
Male	Ref		
Female	3.75	<**0.001**	2.10 to 5.40
**Educational level**			
Less than university	Ref		
University	-0.36	0.657	-1.93 to 1.22
**Health literacy**			
Low	Ref		
High	-3.38	**0.010**	-5.94 to -0.81

S=survey; S1, April 17 to May 14, 2020; S2, May 15 to June 22, 2020; S3, October 30 to December 1st, 2020; S4, June 18, 2021, to December 30, 2021

Model B: Self-reported adherence to government restrictions

	Coefficient	*P-value*	95%CI
10-point increase<br>**Self-reported worry**	1.565	**<0.001**	1.36 to 1.77
**Period**			
S1	Ref		
S2	-5.72	**<0.001**	-7.96 to -3.47
S3	5.97	**<0.001**	3.89 to 8.05
S4	-5.45	**<0.001**	-7.15 to -3.76
**Age**			
18-39	Ref		
40-64	6.71	**<0.001**	5.41 to 8.01
>64	7.39	**<0.001**	5.56 to 9.21
**Gender**			
Male	Ref		
Female	3.43	**<0.001**	2.19 to 4.68
**Educational level**			
Less than university	Ref		
University	0.82	0.177	-0.37 to 2.01
**Health literacy**			
Low	Ref		
High	3.75	**<0.001**	1.81 to 5.68

S=survey; S1, April 17 to May 14, 2020; S2, May 15 to June 22, 2020; S3, October 30 to December 1st, 2020; S4, June 18, 2021, to December 30, 2021

Model C: Perceived adherence of others to government restrictions

	Coefficient	*P-value*	95%CI
10-point increase			
**Self-reported worry**	-0.61	<**0.001**	-0.89 to -0.37
**Period**			
S1	Ref		
S2	-15.71	<**0.001**	-18.31 to-13.11
S3	0.41	0.740	-1.99 to 2.80
S4	-14.95	<**0.001**	-16.94 to -12.96
**Age**			
18-39	Ref		
40-64	4.60	<**0.001**	3.11 to 6.10
>64	7.15	<**0.001**	5.05 to 9.26
**Gender**			
Male	Ref		
Female	-0.59	0.420	-2.04 to 0.85
**Educational level**			
Less than university	Ref		
University	1.54	**0.03**	0.17 to 2.91
**Health literacy**			
Low	Ref		
High	-1.56	0.170	-3.79 to 0.67

S=survey; S1, April 17 to May 14, 2020; S2, May 15 to June 22, 2020; S3, October 30 to December 1st, 2020; S4, June 18, 2021, to December 30, 2021
