# Supplementary material for: Citizen Worry and Adherence in Response to Government Restrictions in Switzerland During the COVID-19 Pandemic: Repeated Cross-Sectional Online Surveys
Source: Interact J Med Res. 2025 Jan 7;14:e55636. doi: 10.2196/55636 (PMC11751645; doi:10.2196/55636)
Supplement: Multimedia Appendix 6 [file ijmr_v14i1e55636_app6.pdf]

Supplementary table 6: Perceived changes of daily life during the first pandemic wave

	**n**	**%**
Physical	684	94
Events	418	58
Commitments	379	52
Home office	355	49
Cancellation of organized trips, self-quarantine or self-isolation	314	43
Not leaving home for several consecutive days	263	36
Missed workdays	171	24
Other changes	59	8
No change	5	1
