# Supplementary material for: Citizen Worry and Adherence in Response to Government Restrictions in Switzerland During the COVID-19 Pandemic: Repeated Cross-Sectional Online Surveys
Source: Interact J Med Res. 2025 Jan 7;14:e55636. doi: 10.2196/55636 (PMC11751645; doi:10.2196/55636)
Supplement: Multimedia Appendix 7 [file ijmr_v14i1e55636_app7.pdf]

Supplementary table 7: Impact of restrictions on daily life, S2, S3, S4

	**S2**		**S3**		**S4**	
	n	%	n	%	n	%
I lost my job or I had to close my business	199	35	280	37	1305	49
I lost part of my income (partial unemployment, reduction of my activity rate, etc.)	176	31	259	34	554	21
I feel less productive	174	31	215	28	486	18
I feel more lonely	134	24	202	26	472	18
I feel isolated	131	23	145	19	414	16
The restrictions currently do not have a significant impact on my life	86	15	61	8	185	7
Other	39	7	6	1	47	2

S=survey; S2, May 15 to June 22, 2020; S3, October 30 to December 1st, 2020; S4, June 18, 2021, to December 30, 2021
