# Supplementary material for: Citizen Worry and Adherence in Response to Government Restrictions in Switzerland During the COVID-19 Pandemic: Repeated Cross-Sectional Online Surveys
Source: Interact J Med Res. 2025 Jan 7;14:e55636. doi: 10.2196/55636 (PMC11751645; doi:10.2196/55636)
Supplement: Multimedia Appendix 8 [file ijmr_v14i1e55636_app8.pdf]

Supplementary table 8: Concerns of respondents, S3, S4

	**S3**		**S4**	
	n	%	n	%
Are you concerned about [yes/mostly yes]				
- very vulnerable people?	698	91	2058	77
- the deterioration of living conditions?	574	75	1929	72
- the economy?	573	75	1668	62
- yourself or your family?	563	73	1552	58
- the deterioration of working conditions?	534	70	1742	65
Are you depressed about the possibility of another wave? [yes/mostly yes]	379	49	1177	44

S=survey; S3, October 30 to December 1st, 2020; S4, June 18, 2021, to December 30, 2021
